# Wheat MYB46-like Transcription Factor Stimulates Cuticular Wax Biosynthesis

**DOI:** 10.3390/biom16060872

**Published:** 2026-06-15

**Authors:** Linzhu Fang, Pengfei Zhi, Jiao Liu, Haoyu Li, Xiaoyu Wang, Cheng Chang

**Affiliations:** College of Life Sciences, Qingdao University, Qingdao 266071, China

**Keywords:** *Triticum aestivum*, cuticular lipid, transcriptional regulation, long-chain acyl-CoA synthetase 1

## Abstract

Cuticular wax mixtures are the major components of the lipophilic cuticle coating of plant aerial organs during primary growth and they protect plants from environmental stresses. Decoding cuticular wax biosynthesis in bread wheat (*Triticum aestivum* L.) could contribute to the genetic improvement of this agriculturally important crop. Herein, we revealed that the wheat MYB46-like transcription factor TaMYB46 positively regulates cuticular wax by activating transcription of the *long-chain acyl-CoA synthetase 1* (*TaLACS1*) gene. Knockdown of the wheat *TaMYB46* gene resulted in significantly reduced cuticular wax loads and increased permeability of the wheat leaf cuticle. Furthermore, wheat long-chain acyl-CoA synthetase TaLACS1 was identified as a core component of the cuticular lipid biosynthetic machinery. Knockdown of the *TaLACS1* gene led to reduced cuticular wax accumulation and increased leaf cuticle permeability. Moreover, the transcription factor *TaMYB46* was found to enrich at the *TaLACS1* promoter regions and activate *TaLACS1* gene transcription. These findings collectively support the conclusion that the transcription factor *TaMYB46* stimulates cuticular wax biosynthesis, likely by activating *TaLACS1* transcription.

## 1. Introduction

As an agriculturally important cereal crop, allohexaploid bread wheat (*Triticum aestivum* L., AABBDD) provides dietary carbohydrates and proteins for humans [1]. Global population growth leads to an increasing demand for wheat grains [2]. However, wheat production is threatened by environmental challenges like drought, salinity, temperature, and ultraviolet stress, which become increasingly severe under climate change [1]. Hydrophobic cuticle coats the plant aerial organs during primary growth and shields plant tissues from the surroundings [3,4,5]. The cuticle not only participates in the plant developmental events, but also plays key roles in the plant adaptation to abiotic and biotic stresses [6,7,8,9]. Decoding the cuticular lipid biosynthesis could contribute to wheat genetic improvement of stress resistance.

Although chemical compositions of the cuticle vary among plant species, cultivars, and organs, and depend on developmental stages and environmental conditions, the lipophilic cuticle is mainly composed of the cutin matrices filled and sealed with wax mixtures [10]. Cutin matrices predominantly comprise crosslinked polyesters of oxygenated long-chain (C16 and C18) fatty acids, whereas cuticular wax mixtures mainly consist of very-long-chain (VLC, >C20) fatty acids, alcohols, aldehydes, alkanes, esters, and ketones [11,12]. As summarized by prior reviews, cuticular wax biosynthesis mainly occurs in the endoplasmic reticulum (ER) of plant epidermal cells [13]. As reported by research in the model plant *Arabidopsis thaliana*, C16 and C18 fatty acids generated in the plastid are first activated by long-chain acyl-coenzyme A synthases (LACS) to form the C16 and C18 acyl-CoAs, the common precursors for the biosynthesis of cuticular wax and cutin [14,15,16]. These long-chain (C16 and C18) acyl-CoAs are elongated by the fatty acid elongase (FAE) and ECERIFERUM2-LIKE proteins to very-long-chain (VLC, >C20) acyl-CoAs [17,18,19,20,21,22,23,24,25]. Cuticular wax components, VLC aldehydes, alkanes, primary and secondary alcohols, ketones, and esters, are modified from VLC acyl-CoAs via either the alkane-forming pathway or alcohol-forming pathway [26,27,28,29,30,31,32,33,34]. Wax mixtures are transported from the ER to the cuticular regions by the conventional Golgi and trans-Golgi network (TGN)-trafficking pathways, ATP-binding cassette (ABC) transporters, and lipid transfer proteins (LTPs) [35,36,37,38,39,40,41,42,43].

As elucidated by studies of *A. thaliana*, cuticular wax biosynthesis is modulated by various regulators, including transcription factors [10,11,12]. For instance, the Arabidopsis MYB-type transcription factors AtMYB30, AtMYB16, AtMYB96, and AtMYB106 positively regulate cuticular wax biosynthesis by activating transcription of wax biosynthesis genes like *AtECR*, *AtCER1* and *AtKCS1* [44,45,46,47]. Similarly, MYB-type transcription factor AtMYB46 could activate transcription of cuticular lipid biosynthesis gene *AtLACS1* and potentiate cuticular wax biosynthesis [48]. It was recently demonstrated that wheat homologs of MYB30, MYB16, MYB96, and MYB106, resembling their counterparts in Arabidopsis, positively regulate wheat cuticular wax biosynthesis [49,50,51]. However, whether and how MYB46-like transcription factor regulates wheat wax biosynthesis remains unknown.

Herein, we aimed to characterize the MYB46-like transcription factor in the regulation of cuticular wax biosynthesis in bread wheat. It was hypothesized that wheat MYB46-like transcription factor, resembling its Arabidopsis homolog, might regulate cuticular wax biosynthesis probably by targeting the wheat *LACS1* homologs. Therefore, in this study we identified the wheat MYB46-like transcription factor and examined its effects on cuticular wax accumulation and determined whether TaMYB46 directly regulates *TaLACS1* transcription.

## 2. Materials and Methods

### 2.1. Wheat Material and Maintenance

Wheat cultivar Yannong 999 was used for the gene transcript accumulation measurement, gene silencing, cuticular lipid composition measurement, cuticle permeability analysis, as well as characterization of protein enrichment at gene promoters, whereas *Arabidopsis* ecotype Col-0 was employed for the gene promoter activation assay. Wheat cultivar Yannong 999 employed in this study was developed by Shandong Yantai Academy of Agricultural Sciences, and was kindly provided by the Qingdao Xinyijia Seed Industry Co., Ltd. (Qingdao, China). The pedigree of cultivar Yannong 999 is Yanhangxuan 2/Lin 9511//Yan BLU14-15, and its released number is Shandong (2011), South region of Huang-Huai (2016), Shanxi (2018). Yannong 999 and Col-0 seedlings were cultivated in a growth chamber under a 16 h light photoperiod, a 21 °C day/18 °C night cycle, and 70% relative humidity. Four wheat seedlings were cultivated in 300 mL pots containing mixtures of nutritive medium and sterile soil (1:1, *v*:*v*). Wheat plants were irrigated with 60 mL of water per pot and fertilized with water-soluble compound fertilizer Huawuque.

### 2.2. Gene Transcript Accumulation Measurement

Transcript accumulation of *TaMYB46* and *TaLACS1* genes in wheat leaves was analyzed by reverse transcription-quantitative polymerase chain reaction (RT-qPCR) assay as previously described [52]. EasyPure Plant RNA kit (Transgenbiotech, Beijing, China), TransScript one-step gDNA removal and cDNA synthesis supermix (Transgenbiotech, Beijing, China), and ABI real-time PCR system with the qPCR Master Mix (Invitrogen, Carlsbad, CA, USA) were employed for this study. *TaMYB46* and *TaLACS1* genes were analyzed using primers 5′CAGGAAACAGCAATGTGG3′/5′GTTGACGATGAGGTCTTC3′ and 5′CAAAGAAGCAGGTTTTCAC3′/5′TGGACGCCATCCAAGCATC3′, respectively. qPCR assay was conducted under the program: 95 °C for 2 min, 40 cycles at 95 °C for 20 s, 56 °C for 20 s, and 72 °C for 15 s, followed by 72 °C for 1 min. Housekeeping gene *TaGADPH* (using primers 5′TTAGACTTGCGAAGCCAGCA3′/5′ AAATGCCCTTGAGGTTTCCC3′) was employed as a reference for RT-qPCR analysis. Calculation of the relative expression levels was achieved using the Bio-Rad CFX Manager 3.1 software (Bio-Rad Laboratories) with the 2^−ΔΔCt^ method. Three technical replicates of the RT-qPCR assay were statistically analyzed using Student’s *t*-test, and similar results were obtained for three biological replicates using independently prepared samples.

### 2.3. Wheat Gene Silencing Assay

*TaMYB46* and *TaLACS1* genes were, respectively, silenced by barley stripe mosaic virus-induced gene silencing (BSMV-VIGS) techniques in wheat leaves as previously described [53]. About 200 bp fragment of *TaMYB46* and *TaLACS1* genes were amplified using primers 5′AAGGAAGTTTAAACTCAACTTGGAAATCAAGG3′/5′AACCACCACCACCGTAGTACTGCTGAGGGCACCAC3′ and 5′AAGGAAGTTTAATACTTTGCTTTGTTCGCAGC3′/5′AACCACCACCACCGTGCATGTCCTTAAAGAGCTCG3′, respectively. BSMV-VIGS wheat plants or leaves were employed for the gene transcript accumulation measurement, cuticular lipid composition characterization, and cuticle permeability analysis. Wheat plants infected with BSMV-*γ* (empty vector) were employed as negative controls and similar results were obtained for three biological replicates using independently prepared samples.

### 2.4. Cuticular Wax Composition Analysis and Cuticle Permeability Measurement

Cuticular wax composition analysis was performed by gas chromatography-mass spectrometry (GC-MS) as previously described [54,55,56]. Cuticular wax mixtures were extracted from wheat leaves using chloroform (Merck, Rahway, NJ, USA), and analyzed using capillary gas chromatography (GC, 5890 Series II, Agilent Technologies) and a flame ionization detector (FID, 6890 N, Agilent Technologies, Santa Clara, CA, USA), with a mass spectrometer (MS, MSD 5973, Agilent Technologies, Santa Clara, CA, USA).   Three technical replicates of the GC-MS assay were statistically analyzed using Student’s *t*-test. Cuticle permeability was analyzed by water loss and chlorophyll leaching assay as previously described [54,55,56]. BSMV-VIGS wheat leaves were dipped in ultrapure water for 1 h in the dark, and the leaves were detached for the water loss and chlorophyll leaching assay as described.

### 2.5. Analysis of Protein Enrichment at Gene Promoters

TaMYB46 protein enrichment at the *TaLACS1* gene promoters was analyzed by chromatin immunoprecipitation-quantitative polymerase chain reaction (ChIP-qPCR) as previously described [54,55]. Coding sequence of *TaMYB46* genes was amplified using the primers 5′GGGGACAAGTTTGTACAAAAAAGCAGGCTTCATGAGGAAGCCCGTGGAGTG3′/5′GGGGACCACTTTGTACAAGAAAGCTGGGTCCTCAACTTGGAAATCAAGGA3′, and cloned into the vector pCAMBIA1300-HA to create the pCAMBIA1300-TaMYB46-HA. Wheat protoplast preparation and transfection, as well as ChIP-qPCR, were conducted as previously described [54,55]. Promoter fragments of *TaLACS1-1A*, *TaLACS1-1B*, and *TaLACS1-1D* genes were analyzed by ChIP-qPCR using primers 5′GAGAATAAACAGTATAATAG3′/5′ACTCGGCATAATAGTCTTAT3′, 5′TTTCAGAAAAGATAGATATG3′/5′CAAACTAGAGGGTGAAGTAG3′, 5′TTGACAGACGACATGATACA3′/5′TTCTCTCGAGACTATCGCGC3′, 5′CAAGAACTTTTCTGTTCCCA3′/5′GAGGAGAAGAAGAAGAACTA3′, 5′CACTCGATCCAAGCCATCTC3′/5′AGATTGCAGGACAGGAAACT3′, and 5′GATGTGCAGAAAAGTACTAC3′/5′CTCCCCTGAATTTATCAATG3′, respectively. Wheat protoplast cells transfected with RNAi-TaMYB46 were employed as negative controls. Three technical replicates of the ChIP-qPCR assay were statistically analyzed using Student’s *t*-test, and similar results were obtained for three biological replicates using independently prepared samples.

### 2.6. Gene Promoter Activation Assay

Activation of the *TaLACS1* gene promoter by the transcription factor TaMYB46 was analyzed by the Dual-Luciferase reporter assay as previously described [48,49]. *TaLACS1-1A*, *TaLACS1-1B*, and *TaLACS1-1D* gene promoter regions were amplified using the primers 5′GGGGACAAGTTTGTACAAAAAAGCAGGCTTCTTTTTCCTGAACAGGTCAGT3′/5′GGGGACCACTTTGTACAAGAAAGCTGGGTCCAAACTAGAGGGTGAAGTAG3′, 5′GGGGACAAGTTTGTACAAAAAAGCAGGCTTCCCGCAAACCGAGGCTCGCGA3′/5′GGGGACCACTTTGTACAAGAAAGCTGGGTCCAAACTAGAGGGTGGAGTAT3′, 5′GGGGACAAGTTTGTACAAAAAAGCAGGCTTCTGACAGACGATATGACAGAA3′/5′GGGGACCACTTTGTACAAGAAAGCTGGGTCTCAATGCATAGCACCGACCA3′, and cloned into the vectors 5XGAL4-LUC to generate reporter constructs. *Arabidopsis* protoplast preparation and transfection, as well as Dual-Luciferase reporter assay, were conducted as previously described. The Dual-Luciferase reporter assay was conducted as described previously [54,55]. Three technical replicates of the Dual-Luciferase reporter assay were statistically analyzed using Student’s *t*-test, and similar results were obtained for three biological replicates using independently prepared samples.

### 2.7. Statistical Analysis

For the analysis of gene transcription, protein enrichment at gene promoter regions, cuticular wax accumulation, and leaf cuticle permeability, at least three independent experiments were performed for each experiment, and wheat leaves or protoplasts were randomly chosen for each group. Three technical replicates were analyzed using Student’s *t*-test, and values represent the mean ± standard deviation (*n. s. p* > 0.05, * 0.01 < *p* < 0.05, ** *p* < 0.01, *n. s.* represents no significant difference). These assays were repeated in three independent biological replicates using independently prepared samples with similar results.

## 3. Results

### 3.1. Identification and Sequence Analysis of Wheat MYB46-like Transcription Factor TaMYB46

**Figure 1:**
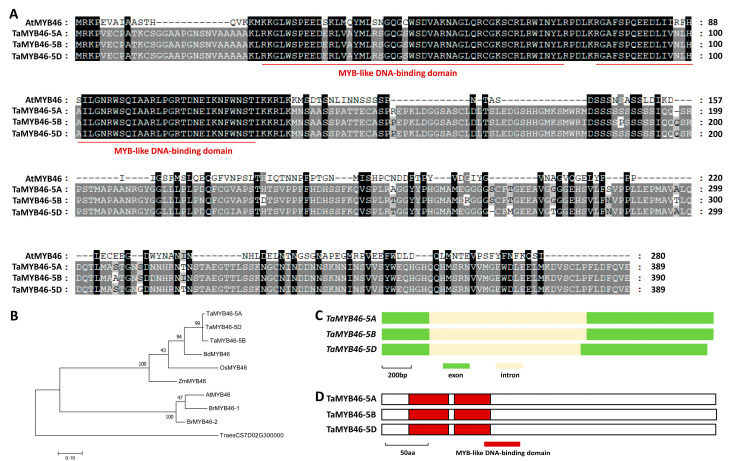
Sequence and phylogenetic analysis of wheat TaMYB46. (**A**) Amino acid sequence comparison of wheat TaMYB46-5A, TaMYB46-5B, TaMYB46-5D, and *Arabidopsis* AtMYB46. Location of conserved MYB-like DNA-binding domain was underlined in red. (**B**) Phylogenetic tree reconstruction using wheat TaMYB46, *Arabidopsis* AtMYB46, mustard BrMYB46, *Brachypodium* BdMYB46, maize ZmMYB46, and rice OsMYB46. A maximum-likelihood tree and 500 bootstrap replicates were employed. (**C**) Intron-exon organization of wheat *TaMYB46* genes. (**D**) Domain arrangement of wheat TaMYB46 proteins.

*Arabidopsis* transcription factor AtMYB46 plays a key role in the regulation of cuticular lipid biosynthesis [48]. Herein, we searched against the wheat protein database using the *Arabidopsis* AtMYB46 (At5g12870) protein sequence as the query and isolated wheat MYB46-like transcription factor TaMYB46. Three highly homologous *TaMYB46* genes, *TaMYB46-5A* (*TraesCS5A02G101000*), *TaMYB46-5B* (*TraesCS5B02G105900*), and *TaMYB46-5D* (*TraesCS5D02G113300*), were separately identified from the wheat 5A, 5B, and 5D chromosomes. Wheat TaMYB46-5A, TaMYB46-5B, TaMYB46-5D and *Arabidopsis* AtMYB46 proteins share 37% amino acid sequence identity (Figure 1A). Phylogenetic tree reconstruction further validated that TaMYB46-5A, TaMYB46-5B, and TaMYB46-5D are the wheat homologs of *Arabidopsis* AtMYB46 (Figure 1B). Two exons and one intron exist in the genome sequence of wheat *TaMYB46-5A*, *TaMYB46-5B*, and *TaMYB46-5D* genes (Figure 1C). Two MYB-like DNA-binding domains were identified from the N-terminal parts of wheat TaMYB46-5A, TaMYB46-5B, and TaMYB46-5D proteins (Figure 1D).

### 3.2. Functional Characterization of TaMYB46 in Wheat Cuticular Wax Biosynthesis

**Figure 2:**
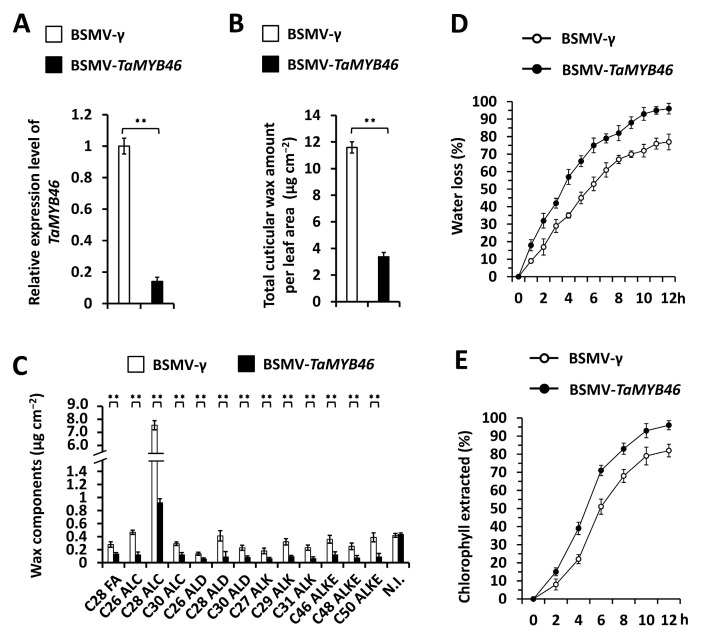
Functional characterization of wheat *TaMYB46* genes in cuticular wax biosynthesis. (**A**) Relative accumulation of *TaMYB46* gene transcripts in the wheat leaves with silenced *TaMYB46* genes. (**B**) Loads of cuticular wax on the wheat leaves with knockdown of *TaMYB46* genes. (**C**) Amounts of wax FA (fatty acid), ALC (alcohol), ALD (aldehyde), ALK (alkane), ALKE (alkyl ester), and N.I. (not identified compound) in the wheat leaves with silenced *TaMYB46* genes. (**D**) Water loss and (**E**) chlorophyll extraction levels were analyzed in wheat leaves with silenced *TaMYB46* genes. For (**A**–**E**), data were statistically analyzed using Student’s *t*-test, and significant difference was represented by ** (*p* < 0.01).

To study the potential roles of the transcription factor TaMYB46 in the regulation of wheat cuticular wax biosynthesis, we conducted the BSMV-VIGS assay to silence all endogenous *TaMYB46* genes in the wheat cultivar Yannong 999 plants. As shown in Figure 2A, the accumulation level of the *TaMYB46* gene transcript was significantly reduced in the leaves of wheat BSMV-*TaMYB46* plants, compared with the leaves of BSMV-*γ* control plants. Accumulation of cuticular wax on these wheat leaves was determined by the GC-MS assay. As shown in Figure 2B, silencing of *TaMYB46* (BSMV-*TaMYB46*) gene led to the reduction in cuticular wax accumulation to 29.2%. The amounts of major cuticular wax components, including VLC alcohols (ALCs), aldehydes (ALDs), fatty acids (FAs), alkanes (ALKs), alkyl esters (ALKEs), were significantly decreased in the leaves of wheat BSMV-*TaMYB46* plants, compared with the leaves of BSMV-*γ* control plants (Figure 2C). These data suggested that the wheat transcription factor TaMYB46 positively regulates biosynthesis of cuticular lipid wax.

We analyzed the potential regulation of TaMYB46 on the wheat leaf cuticle permeability. As shown in Figure 2D,E, wheat leaves with a silenced *TaMYB46* gene displayed enhanced rates of water loss and chlorophyll leaching compared with the leaves of BSMV-*γ* control plants, suggesting that transcription factor TaMYB46 negatively regulates wheat leaf cuticle permeability. Collectively, these findings imply that wheat transcription factor TaMYB46 potentiates biosynthesis of cuticular wax, positively contributing to the wheat leaf cuticle barrier property.

### 3.3. Identification and Sequence Analysis of Wheat TaLACS1

**Figure 3:**
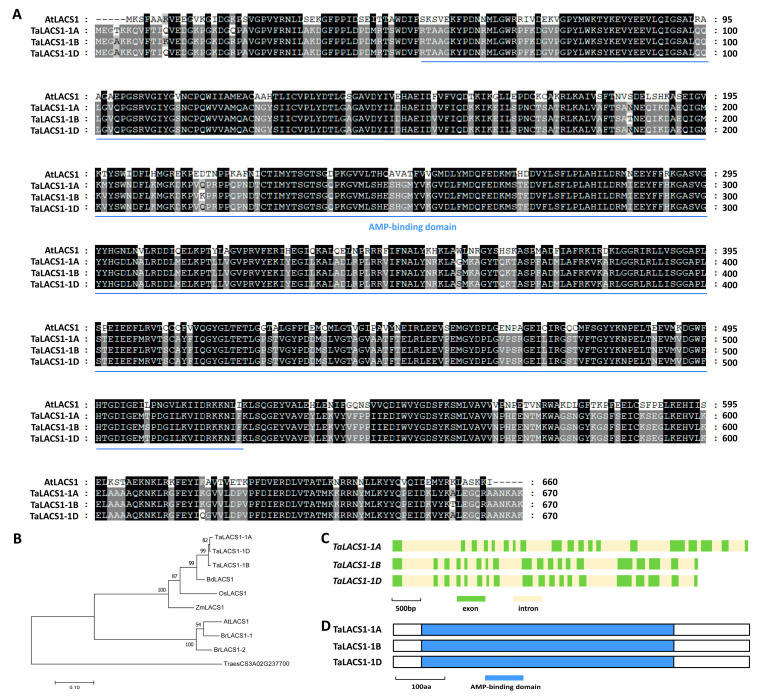
Sequence and phylogenetic analysis of wheat TaLACS1. (**A**) Amino acid sequence comparison of wheat TaLACS1-1A, TaLACS1-1B, TaLACS1-1D, and *Arabidopsis* AtLACS1. Location of conserved AMP-binding domain is underlined in red. (**B**) Phylogenetic tree reconstruction using wheat TaLACS1, *Arabidopsis* AtLACS1, mustard BrLACS1, *Brachypodium* BdLACS1, maize ZmLACS1, and rice OsLACS1. A maximum-likelihood tree and 500 bootstrap replicates were employed. (**C**) Intron-exon organization of wheat *TaLACS1* genes. (**D**) Domain arrangement of wheat TaLACS1 proteins.

*Arabidopsis* long-chain acyl-CoA synthetase 1 (AtLACS1) functions as an essential component of cuticular lipid biosynthetic machinery [15]. Herein, we searched against the wheat protein database using the *Arabidopsis* AtLACS1 (At2g47240) protein sequence as the query and isolated the wheat long-chain acyl-CoA synthetase TaLACS1. Three highly homologous *TaLACS1* genes, *TaLACS1-1A* (*TraesCS1A02G075000*), *TaLACS1-1B* (*TraesCS1B02G093600*), and *TaLACS1-1D* (*TraesCS1D02G077600*), were separately identified from the wheat 1A, 1B, and 1D chromosomes. Wheat TaLACS1-1A, TaLACS1-1B, TaLACS1-1D, and *Arabidopsis* AtLACS1 proteins share 62% amino acid sequence identity (Figure 3A). Phylogenetic tree reconstruction further validated that TaLACS1-1A, TaLACS1-1B, and TaLACS1-1D are wheat homologs of *Arabidopsis* AtLACS1 (Figure 3B). Nineteen exons and eighteen introns exist in the genome sequence of wheat *TaLACS1-1A*, *TaLACS1-1B*, and *TaLACS1-1D* genes (Figure 3C). One AMP-binding domain was identified from TaLACS1-1A, TaLACS1-1B, and TaLACS1-1D proteins (Figure 3D).

### 3.4. Functional Characterization of TaLACS1 in Wheat Cuticular Wax Biosynthesis

**Figure 4:**
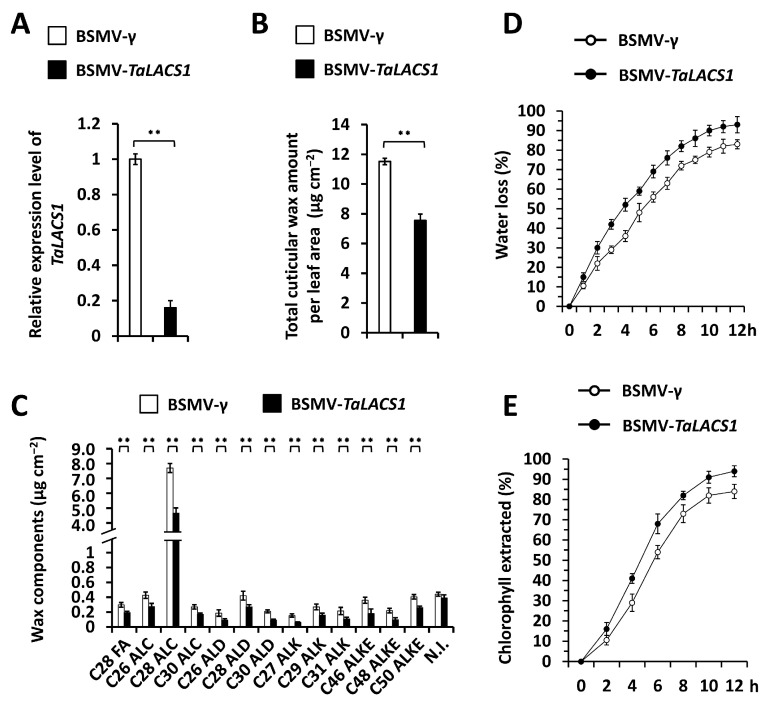
Functional characterization of wheat *TaLACS1* genes in cuticular wax biosynthesis. (**A**) Relative accumulation of the *TaLACS1* gene transcripts in the wheat leaves with silenced *TaLACS1* gene. (**B**) Loads of cuticular wax on the wheat leaves with knockdown of the *TaLACS1* gene. (**C**) Amounts of wax components in the wheat leaves with silenced *TaLACS1* gene. (**D**) Water loss and (**E**) chlorophyll extraction levels were analyzed in wheat leaves with silenced *TaLACS1* gene. For (**A**–**E**), data were statistically analyzed using Student’s *t*-test, and significant difference was represented by ** (*p* < 0.01).

To explore the potential function of long-chain acyl-CoA synthetase TaLACS1 in the biosynthesis of the wheat cuticular lipid, we employed the BSMV-VIGS assay to silence all endogenous *TaLACS1* genes in the wheat cultivar Yannong 999 plants. As shown in Figure 4A, the accumulation level of the *TaLACS1* gene transcript was significantly reduced in the leaves of wheat BSMV-*TaLACS1* plants, compared with the leaves of BSMV-*γ* control plants. Accumulation of cuticular wax on these wheat leaves was determined by the GC-MS assay. As shown in Figure 4B, silencing of *TaLACS1* (BSMV-*TaLACS1*) gene led to the reduction in cuticular wax accumulation to 65.5%. Amounts of major cuticular wax components, including VLC ALCs, ALDs, FAs, ALKs, and ALKEs, were significantly decreased in the leaves of wheat BSMV-*TaLACS1* plants, compared with the leaves of BSMV-*γ* control plants (Figure 4C). These data suggested that the wheat long-chain acyl-CoA synthetase TaLACS1 plays a key role in the biosynthesis of wheat cuticular wax. We analyzed the potential effect of TaLACS1 on the wheat leaf cuticle permeability. As shown in Figure 4D,E, wheat leaves with a silenced *TaLACS1* gene displayed enhanced rates of water loss and chlorophyll leaching compared with the leaves of BSMV-*γ* control plants, suggesting that long-chain acyl-CoA synthetase TaLACS1 negatively regulates wheat leaf cuticle permeability. These findings collectively implicate that wheat long-chain acyl-CoA synthetase TaLACS1 functions as an essential component of cuticular wax biosynthetic machinery and positively contributes to the wheat leaf cuticle barrier property.

### 3.5. Regulation Analysis of TaLACS1 Gene Transcription by TaMYB46

**Figure 5:**
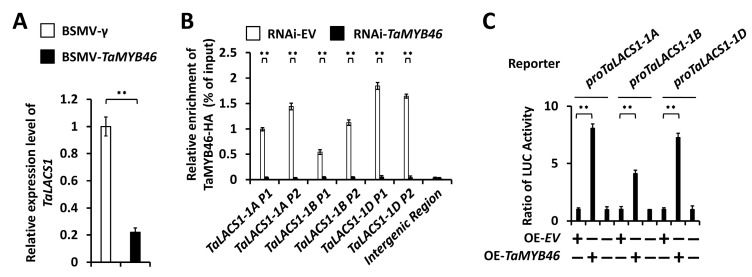
Analyses of TaMYB46 regulation on *TaLACS1* gene transcription. (**A**) Relative accumulation of *TaLACS1* gene transcripts in the wheat leaves with silenced *TaMYB46* genes. (**B**) Measurement of *TaMYB46* enrichment at *TaLACS1* promoter regions. P1 and P2 represent two fragments of the indicated gene promoter regions. (**C**) Activation of *TaLACS1* promoters by TaMYB46. For (**A**–**C**), data were statistically analyzed using Student’s *t*-test, and significant difference was represented by ** (*p* < 0.01).

In the dicot model plant *Arabidopsis*, transcription factor AtMYB46 plays a key role in the regulation of cuticular lipid biosynthesis [51]. To examine the potential regulation of wheat transcription factor TaMYB46 on the expression of cuticular lipid biosynthesis gene *TaLACS1*, we employed the BSMV-VIGS technique to silence all endogenous *TaMYB46* genes in the wheat cultivar Yannong 999 plants and analyzed the accumulation level of the *TaLACS1* gene transcript using RT-qPCR. As shown in Figure 5A, the accumulation level of the *TaLACS1* gene transcript was significantly reduced in the leaves of wheat BSMV-*TaMYB46* plants, compared with the leaves of BSMV-*γ* control plants, suggesting that the transcription factor TaMYB46 positively regulates the expression of cuticular lipid biosynthesis gene *TaLACS1.* We asked whether wheat transcription factor TaMYB46 enriches at the promoter region of cuticular lipid biosynthesis gene *TaLACS1*. To address this question, we transfected the wheat protoplast cells with the *TaMYB46-HA* construct and examined the enrichment of TaMYB46-HA at the promoter region of *TaLACS1 genes* using a ChIP-qPCR assay. As shown in Figure 5B, DNA fragments of *TaLACS1-1A*, *TaLACS1-1B*, and *TaLACS1-1D* gene promoters were found to be immunoprecipitated with the antibody against TaMYB46-HA, suggesting that the wheat transcription factor TaMYB46 occupies the promoter regions of the *TaLACS1* gene. To examine the potential regulation of wheat transcription factor TaMYB46 on the transcription driven by *TaLACS1* gene promoters, we conducted the Dual-Luciferase reporter assay using the *Arabidopsis* protoplast cells. In the presence of transcription factor TaMYB46, relative activity of *LUC* reporter gene driven by the *TaLACS1* gene promoters increased to above 4.1 compared with 1 for the empty vector control, suggesting that wheat transcription factor TaMYB46 activates the transcription driven by *TaLACS1* gene promoters (Figure 5C). Collectively, these findings suggest that wheat transcription factor TaMYB46 activates the transcription of cuticular lipid biosynthesis gene *TaLACS1* to stimulate cuticular wax biosynthesis.

## 4. Discussion

### 4.1. Wheat Transcription Factor TaMYB46 Potentiates Cuticular Wax Accumulation

In the *A. thaliana*, transcription factor AtMYB46 plays a key role in the regulation of cuticular lipid biosynthesis [48]. *Arabidopsis MYB46* mutants, particularly the *myb46-4* (exon T-DNA insertion mutant), exhibited reduced cuticular lipid accumulation and increased cuticle permeability. In contrast, overexpression of AtMYB46 resulted in the reinforced cuticle integrity, increased accumulation of cuticular wax, as well as improved drought tolerance [48]. Herein, we demonstrated that wheat MYB46-like transcription factor TaMYB46 stimulates cuticular wax biosynthesis. Knock-down of wheat *TaMYB46* genes resulted in significantly reduced loads of cuticular wax and increased cuticle permeability. These studies support that the positive regulation of MYB46-like transcription factor on cuticular lipid biosynthesis is conserved between the dicot *Arabidopsis* and the monocot bread wheat.

A plethora of myeloblastosis (MYB) transcription factors were identified to regulate cuticular lipid biosynthesis in the model plant *A. thaliana* and crop plant *T. aestivum* [44,45,46,47,48,49,50,51,56,57,58,59]. *Arabidopsis* R2R3-type MYB transcription factors AtMYB96, AtMYB94, AtMYB30, AtMYB60, AtMYB46, AtMYB49, AtMYB16, and AtMYB106 positively regulate biosynthesis of cuticular wax [44,45,46,47,48,57,58,59]. Similarly, wheat MYB transcription factors TaEPBM1/TaMYB96, TaMYB30, TaMYB60, TaMIXTA1, TaMIXTA2, and TaMYB49 stimulate biosynthesis of cuticular lipids [49,50,51,56]. Accumulating evidence revealed that the cuticular lipid biosynthesis is regulated by the developmental signals and environmental stimuli [7]. Therefore, it is intriguing to examine the potential roles and interplays of these MYB transcription factors in the regulation of cuticular wax biosynthesis in response to the developmental and environmental cues.

### 4.2. Wheat Long-Chain Acyl-CoA Synthetase TaLACS1 Is Essential for Cuticular Wax Biosynthesis

Nine long-chain acyl-CoA synthetases family members were identified from *A. thaliana* [14,15,16]. *Arabidopsis* AtLACS1 is involved in cuticular wax biosynthesis by modifying VLC fatty acids, whereas AtLACS2 plays a key role in the cutin biosynthesis [14,15,16]. SlLACS1, tomato ortholog of AtLACS1, was also identified as an essential component of cuticular wax biosynthesis. The cuticular wax deposition on tomato leaves was remarkably reduced in the *SlLACS1*-knockout plants, whereas cuticle permeability of tomato leaves was significantly enhanced in *SlLACS1*-knockout lines [60]. Similarly, heterologous overexpression of apple *MdLACS1* in *Arabidopsis* resulted in the cuticular wax overaccumulation [61]. In this study, we characterized the function of wheat long-chain acyl-CoA synthetase TaLACS1 in cuticular wax biosynthesis. Accumulation levels of total cuticular wax mixtures, as well as major cuticular wax components like VLC ALCs, ALDs, FAs, ALKs, and ALKEs, were significantly decreased in the wheat leaves with silenced *TaLACS1* gene. Consistent with this, water loss and chlorophyll extraction assays indicated that cuticle permeability was elevated in *TaLACS1*-silenced wheat leaves. These findings suggested that, although cuticular wax compositions vary among plant species, the *LACS1* gene plays an evolutionarily conserved function in the cuticular wax biosynthesis in the dicots and monocots.

### 4.3. Wheat Transcription Factor TaMYB46 Stimulates Cuticular Wax Biosynthesis by Activating TaLACS1 Gene Expression

*Arabidopsis* transcription factor AtMYB46 directly activated cuticular lipid biosynthesis genes like *AtLACS1*, *AtLACS2*, *AtKCS1*, *AtKCS19*, and *AtCER1* to stimulate the cuticle biosynthesis [48]. In this study, we showed that the accumulation level of the *TaLACS1* gene transcript was significantly reduced in the leaves of *TaMYB46*-knockdown wheat plants. The ChIP-qPCR assay revealed that wheat transcription factor TaMYB46 occupies the promoter regions of the *TaLACS1* gene, and Dual-Luciferase reporter assay implicated that wheat transcription factor TaMYB46 activates the transcription driven by the *TaLACS1* gene promoters. These data suggested that wheat transcription factor TaMYB46, resembling its *Arabidopsis* homolog, directly activates the cuticular lipid biosynthesis gene *TaLACS1* to stimulate cuticular wax biosynthesis. Silencing of *TaMYB46* (BSMV-*TaMYB46*) gene led to the reduction in cuticular wax ac-cumulation to 29.2% (Figure 2B), while silencing of *TaLACS1* (BSMV-*TaLACS1*) gene led to the reduction in cuticular wax accumulation to 65.5% (Figure 4B), suggesting that TaMYB46 might target other wax biosynthesis genes. Therefore, it is intriguing to examine the potential regulation of TaMYB46 on the transcription of other wax biosynthesis genes like *TaKCS1*, *TaKCS19*, and *TaCER1* in future research.

Through directly activating wax or cutin biosynthesis genes, several wheat transcription factors have been demonstrated to stimulate cuticle biosynthesis [49,50,51]. For instance, wheat MYB transcription factor TaEPBM1 could recognize the *MBS* and *Motif 1* cis-elements to bind to the *TaECR* and *TaCER3* promoters and activate transcription of *TaECR* and *TaCER3* genes, promoting wheat cuticular wax biosynthesis [49]. Similarly, wheat MYB transcription factor TaMYB30 could also recognize the *MBS* and *Motif 1* cis-elements to bind to the *TaKCS2* promoters and stimulate *TaKCS2* transcription, leading to the potentiated wheat cuticular wax biosynthesis [50]. Wheat AP2-type transcription factor TaSHN1 could recognize the *GCC-box* cis-elements to bind to the *TaCYP86A2* and *TaCYP86A4* promoters and activate *TaCYP86A2* and *TaCYP86A4* transcription, stimulating wheat cutin biosynthesis [52]. In this study, we demonstrated that the wheat transcription factor TaMYB46 directly activates the cuticular lipid biosynthesis gene *TaLACS1* to stimulate cuticular wax biosynthesis. Identifying the cis-elements directly recognized by the transcription factor TaMYB46 could provide more insight into the underlying regulatory mechanism in future research.

The waxy cuticle shields plant tissues from the surroundings during the primary growth and facilitates plant resistance against a plethora of abiotic stresses, including water-deficit, salt stress, heat, cold, and ultraviolet B radiation [7]. Cuticular wax-associated traits like increased leaf wax *n*-alkane concentration were found to be correlated with increased productivity in bread wheat [62]. In this study, we identified transcription factor TaMYB46 and long-chain acyl-CoA synthetase TaLACS1 as positive regulators and essential components of wheat cuticular wax biosynthetic machineries, respectively. As proposed by prior reviews, genetically manipulating cuticle biosynthesis genes using new developed genome editing techniques like CRISPR-Cas9 (clustered regularly interspaced short palindromic repeats-CRISPR associated 9) system, targeted mutation breeding techniques such as targeting induced local lesions (TILLING), or cross breeding approaches like marker-assisted selection (MAS), could facilitate wheat breeding for stress resistance [63,64,65,66,67]. Therefore, genetically manipulating *TaMYB46* and *TaLACS1* genes using these advanced techniques might represent a new approach for wheat stress resistance breeding in the future.

In this study, we characterized the function of *TaMYB46* and *TaLACS1* genes in wheat cuticular wax biosynthesis using the BSMV-VIGS technique, generating stable wheat mutants or overexpressing plants of these genes by CRISPR-Cas9 or TILLING might provide more insight into their action mechanisms in future research. In addition, the large-scale genotype analysis of *TaMYB46* and *TaLACS1* genes in global wheat cultivars and identifying potential Elite haplotypes of these genes suitable for the wheat breeding for stress resistance might contribute to the wheat genetic improvement in future research.

## 5. Conclusions

In this study, we characterized the MYB46-like transcription factor in the regulation of cuticular wax biosynthesis in bread wheat and found that the wheat MYB46-like transcription factor TaMYB46 stimulates cuticular wax biosynthesis by activating TaLACS1 *gene* transcription. Silencing of the wheat *TaMYB46* and *TaLACS1* genes attenuates cuticular wax accumulation. Transcription factor TaMYB46 could enrich at *TaLACS1* promoter regions and activate *TaLACS1* gene transcription. These findings collectively support that transcription factor TaMYB46 stimulates cuticular wax biosynthesis by activating *TaLACS1* gene transcription, and provide important information for the genetic improvement of cuticle-associated traits in bread wheat.

## Data Availability

The original contributions presented in this study are included in the article. Further inquiries can be directed to the corresponding author.
